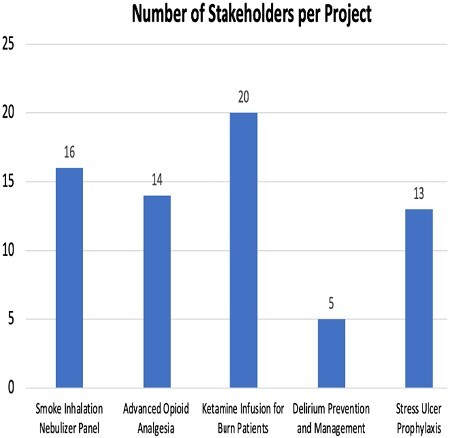# 587 Pharmacy Residents Have a Significant Impact on Process Improvement at Verified Burn Center

**DOI:** 10.1093/jbcr/irae036.221

**Published:** 2024-04-17

**Authors:** Megan Lauzon, Caroline Ko, Sherman Lau, Vina Vargas, Lea Zaballero, Yvonne L Karanas, Clifford C Sheckter

**Affiliations:** Santa Clara Valley Medical Center, San Jose, CA; Stanford/Santa Clara Valley Medical Center, San Jose, CA; Santa Clara Valley Medical Center, San Jose, CA; Stanford/Santa Clara Valley Medical Center, San Jose, CA; Santa Clara Valley Medical Center, San Jose, CA; Stanford/Santa Clara Valley Medical Center, San Jose, CA; Santa Clara Valley Medical Center, San Jose, CA; Stanford/Santa Clara Valley Medical Center, San Jose, CA; Santa Clara Valley Medical Center, San Jose, CA; Stanford/Santa Clara Valley Medical Center, San Jose, CA; Santa Clara Valley Medical Center, San Jose, CA; Stanford/Santa Clara Valley Medical Center, San Jose, CA; Santa Clara Valley Medical Center, San Jose, CA; Stanford/Santa Clara Valley Medical Center, San Jose, CA

## Abstract

**Introduction:**

Quality and process improvement are vital for burn centers to maintain patient safety, systematically review complications, and adopt new practices involving technology, medications, and staff development. The pharmacy is involved in every facet of burn care from the intensive care unit to the operating room. Pharmacy residents participate in quality and process improvement projects during their training, but little published data exists on how pharmacy residents contribute to overall process improvement in a verified burn center.

**Methods:**

Postgraduate Year-Two (PGY2) pharmacy residents specializing in critical care rotate through the Burn Intensive Care Unit (BICU) for 6 weeks. In addition to patient monitoring, advising, and multidisciplinary rounding, pharmacy residents develop a process improvement project for the burn center. Projects are determined in collaboration with the burn unit director, nursing management, and senior ICU pharmacist; agreement is decided with a Delphi process. After drafting, the project is presented and approved by the county hospital’s critical care sub-committee. Following completed sign-off by stakeholders, the project is presented to the Pharmacy and Therapeutics committee for official approval. Pharmacy residents spearhead implementation of the projects by further building out the electronic medical record interface.

We reviewed all PGY-2 Pharmacy Resident led process improvement projects from 2020 to 2023. The primary end point was the total number of projects and their topics. Secondary outcomes included the number of stakeholders per project along with success of each project which was determined by utilization endpoints

**Results:**

There were a total of five projects completed over the 3-year period including: 1) smoke inhalation nebulizer panel, 2) advanced opioid analgesia, 3) stress ulcer prophylaxis panel for major burn patients , 4) delirium prevention and management, and 5) ketamine infusion for pain management. Since implementation, the burn inhalation panel has been used in 4/4 patients with confirmed smoke inhalation injury, the advanced opioid analgesia in 10/11 patients, stress ulcer prophylaxis panel in 17/17 patients, and delirium prevention and management in 91/91 patients. The ketamine infusion for pain management was recently approved and not yet live in the electronic health record.

**Conclusions:**

Pharmacy residents are an innovative and potentiality underutilized resource for the development and implementation of pertinent process improvement projects at burn centers.

**Applicability of Research to Practice:**

Other burn centers may wish to consider the benefits of pharmacy residents within the burn unit for targeted quality and process improvement projects.